# B3GALT4 remodels the tumor microenvironment through GD2-mediated lipid raft formation and the c-met/AKT/mTOR/IRF-1 axis in neuroblastoma

**DOI:** 10.1186/s13046-022-02523-x

**Published:** 2022-10-25

**Authors:** Yong-Liang Sha, Yun Liu, Jia-Xing Yang, Yang-Yang Wang, Bao-Cheng Gong, Yan Jin, Tong-Yuan Qu, Fan-Tong Xia, Lei Han, Qiang Zhao

**Affiliations:** 1 Department of Pediatric Oncology, Tianjin Medical University Cancer Institute and Hospital, National Clinical Research Center for Cancer, Key Laboratory of Cancer Prevention and Therapy, Tianjin’s Clinical Research Center for Cancer, Tianjin, China; 2 Cancer Molecular Diagnostics Core, Tianjin Medical University Cancer Institute and Hospital, National Clinical Research Center for Cancer, Key Laboratory of Cancer Prevention and Therapy, Tianjin’s Clinical Research Center for Cancer, Tianjin, China

**Keywords:** Ganglioside GD2, Lipid raft, Beta-1,3-galactosyltransferase-4, CD8^+^ T-cell chemokine, Immunotherapy, Neuroblastoma

## Abstract

**Background:**

Beta-1,3-galactosyltransferase-4 (B3GALT4) plays a critical regulatory role in tumor biology. However, the role of B3GALT4 in modulating the tumor microenvironment (TME) of neuroblastoma (NB) remains unknown.

**Methods:**

Public datasets and clinical NB samples were collected to evaluate the expression and clinical significance of GD2 and B3GALT4 in NB patients. CCK-8, colony formation, and transwell assays and experiments in tumor-bearing mouse models were conducted to investigate the function of B3GALT4. Flow cytometry, ELISA, immunohistochemistry, immunofluorescence, western blotting, and chemotaxis assays were conducted to ascertain the immunomodulatory mechanism of B3GALT4. The combined therapeutic effect of the lipid raft inhibitor MβCD and anti-GD2 mAb was validated in a murine model of NB.

**Results:**

GD2 was overexpressed in NB tissues and high expression of GD2 was associated with poor prognosis in NB patients. B3GALT4 was downregulated in NB tissues, and low expression of B3GALT4 indicated poor prognosis in NB patients. Silencing B3GALT4 significantly enhanced tumor progression both in vitro and in vivo. Meanwhile, the overexpression of B3GALT4 increased the recruitment of CD8^+^ T lymphocytes via the chemokines CXCL9 and CXCL10. Additionally, B3GALT4 regulated NB-cell GD2 expression and lipid raft formation. Mechanistically, B3GALT4 regulated the expression of CXCL9 and CXCL10 via the c-Met signaling in the lipid rafts and the downstream AKT/mTOR/IRF-1 pathway. The lipid raft inhibitor, MβCD, attenuated B3GALT4 deficiency-induced tumor progression and immune evasion. Last, MβCD combined with anti-GD2 mAb treatment significantly enhanced the antitumor effect and the infiltration of CD8^+^ T cells.

**Conclusions:**

Upregulation of B3GALT4 promotes the secretion of CXCL9 and CXCL10 to recruit CD8^+^ T lymphocytes via the GD2-mediated lipid rafts and the c-Met/AKT/mTOR/IRF-1 pathway. Moreover, lipid raft inhibitors may enhance the efficacy of anti-GD2 immunotherapy for NB.

**Supplementary Information:**

The online version contains supplementary material available at 10.1186/s13046-022-02523-x.

## Background

Neuroblastoma (NB), one of the most common malignant tumors in children, accounts for 10–15% of all pediatric cancer-related deaths [1]. Several anti-GD2 monoclonal antibodies (mAbs) are emerging in the treatment of high-risk NB and have achieved certain clinical effects. However, more than 40% of NB patients fail to respond or develop resistance to anti-GD2 immunotherapy [2]. Therefore, an investigation of the molecular mechanisms involved in therapeutic failure and the development of innovative alternative treatment methods are needed.

The tumor microenvironment (TME) is made up of various cell types and extracellular components. Emerging evidence suggests that the frequency of CD8^+^ T cells in tumors is closely associated with the prognosis and resistance to immunotherapy of high-risk NB patients [3, 4]. Chemokines are a family of small heparin-binding proteins that mediate immune cell trafficking [5]. Among these chemokines, the tumor cell-derived chemokines CXCL9 and CXCL10 are critical in attracting CD8^+^ T lymphocytes to tumor tissues [6, 7]. However, the biological mechanisms of chemokine expression in NB cells are not yet known.

Disialoganglioside GD2 is highly expressed in neuro-ectoderm-related cancer cells and has limited expression in normal tissues. It is a well-suited target for immunotherapy [8]. Beta-1,3-galactosyltransferase-4 (B3GALT4), an important glycosyltransferase involved in ganglioside GD2 synthesis, plays an oncogenic role in multiple cancers [9, 10]. In NB cells, overexpressed B3GALT4 inhibited cell migration [11]. However, the function and mechanism of B3GALT4 in NB immunity remain unclear. Lipid rafts, a particular membrane microdomain type composed of gangliosides, act as important functional platforms for signal transduction [12]. Moreover, the activation of lipid rafts depends mainly on interactions with the gangliosides [13]. A recent study demonstrated that GD2 in glycolipid-enriched microdomains/rafts (GEM/rafts) enhanced the malignant phenotypes of melanomas [14], so it is possible that ganglioside GD2 influences the activation of lipid rafts. The lipid raft-related AKT pathway and c-Met signaling are hyperactivated in various tumors [15–17]. Meanwhile, some studies have demonstrated that PI3K/AKT pathway activation in cancer cells can suppress CD8^+^ T-cell infiltration into tumor tissues [18, 19]. Therefore, we hypothesize that ganglioside GD2 may regulate the tumor microenvironment by signaling through a complex of lipid rafts.

Herein, we found that B3GALT4 was remarkably downregulated in NB tissues and that overexpression of B3GALT4 exhibited the antitumor functions in vitro and in vivo. Meanwhile, we showed that B3GALT4 regulated the lipid raft formation through GD2. Mechanistically, elevated B3GALT4 levels upregulated CXCL9 and CXCL10 expression to recruit CD8^+^ T cells by the c-Met signaling in the lipid rafts and the downstream AKT/mTOR/IRF-1 pathway. Moreover, the combination of a lipid raft inhibitor and anti-GD2 antibody effectively enhanced the therapeutic efficacy of anti-GD2 immunotherapy in murine models. Taken together, our findings may be valuable for understanding the relationship between GD2 and the tumor immune microenvironment and improving the clinical outcome of patients receiving anti-GD2 immunotherapy.

## Materials and methods

### Clinical samples

A total of 81 NB and 25 ganglioneuroma (GN) tumor tissue samples were collected from February 2014 to April 2019 at Tianjin Medical University Cancer Institute and Hospital. The clinical, prognostic, and pathological information of patients was included. The guarantees were fully informed and consented to the research. The study’s procedures were authorized by the Research Ethics Committee of Tianjin Medical University Cancer Institute and Hospital (E20210027) and conducted according to the Code of Ethics of the World Medical Association (Declaration of Helsinki). The clinical characteristics of the patients included in this study are shown in Table S1.

### Bioinformatics analysis and gene set enrichment analysis

The gene expression data and clinical information for GSE49710 [20], GSE85047 [21], and TARGET-NB were downloaded from the GEO database and the TARGET database. There were 498, 283, and 154 patients in each dataset respectively. Additionally, the GSE112447 and GSE90689 datasets were retrieved to analyze the GD2 molecular phenotype. The relationship between B3GALT4 expression, CD8A expression, and INSS stages was determined. Kaplan-Meier and log-rank tests were used to determine the relationship between B3GALT4 expression levels and NB prognosis. Pearson correlation coefficients were used to assess associations between interferon regulatory factor-1 (IRF-1), CXCL9, and CXCL10 expression. A comprehensive analysis of the mRNA expression levels of genes from the GSE49710 dataset was conducted and categorized. To identify the B3GALT4-mediated biological function in NB progression, gene set enrichment analysis (GSEA) of the GSE49710 dataset was performed. A false discovery rate < 0.25 and *P* < 0.05 were considered statistically significant for the enriched gene sets. The R software (version 3.3.2) was used to conduct all bioinformatics and statistical analyses.

### Immunohistochemistry (IHC)

Briefly, histopathological sections (4 μm thickness) were baked at 60 °C overnight and then deparaffinized and dehydrated. Antigen repair and blocking of endogenous peroxidase activity were performed. After that, these slices were incubated with various primary antibodies for 24 h at 4 °C in a damp container and then incubated with matching secondary antibodies for 1 h at ambient temperature. Finally, Diaminobenzidine (DAB) was utilized for 5 min until a brown reaction result was observed. Digital images were acquired using a light microscope. Anti-GD2, anti-B3GALT4, anti-CD8, anti-caveolin-1, anti-CXCL9, and anti-CXCL10 were used as primary antibodies (Table S2).

The results of the IHC assays were analyzed by two professional pathologists who were blinded to the experimental procedures. The frequency of positive cells was classified into five categories: 0 (negative), 1 (1–25%), 2 (26–50%), 3 (51–75%), and 4 (76–100%). The staining degree was categorized as follows: 0 (negative), 1 (weak), 2 (moderate), and 3 (strong). The IHC score was estimated by multiplying the two outcomes mentioned above. The CD8A-positive cells in 200× photographs of tumor tissues were identified to establish the frequency of CD8^+^ T cells.

### Cell culture

The NB cell lines 9464D and 975A2 (gifts from Dr. Rimas Orentas at Seattle Children’s Research Institute) were cultured in high-glucose dulbecco’s modified eagle’s medium (DMEM) containing 10% fetal bovine serum (FBS, BI) and 1% P/S (Gibco) at 37 °C, in a 5% CO_2_ humidified incubator. PCR was used to ensure that the cells were free of mycoplasma infection every month. After freezing, the cells were employed in 20 passages and cultivated for less than 6 months. The cell lines were described in previous publications [22].

### Establishment of stable cell lines for B3GALT4 knockdown and overexpression

ShRNA targeting B3GALT4 was synthesized and inserted into the pLKO.1-puromycin lentiviral vector to generate the B3GALT4 knockdown vector (sh-B3GALT4). The scrambled shRNA was inserted into the pLKO.1-puromycin lentiviral vector and defined as a negative control (Scramble). The shRNA sequences are shown in Table S3. The pCDHO-P-FLAG-Puro lentiviral vector encoding mouse B3GALT4 was constructed by RiboBio (Guangzhou, China) and defined as B3GALT4. An empty vector was defined as a negative control (Vector). The cloned plasmids and packaging plasmids (pMDLg/pRRE, pRSV-Rev, and pMD2.G) were transfected into 293 T cells to synthesize the lentiviral particles used to infect NB cells. Stably transfected 9464D and 975A2 cells were selected with puromycin (4 μg/ml and 3 μg/ml) for 48 h, and then the expression levels of B3GALT4 were assessed by qRT-PCR and western blot.

### Lipid raft inhibitor treatment

To explore the effect of lipid rafts on B3GALT4-mediated biological functions, 9464D cells transfected with sh-B3GALT4 were treated with the lipid raft inhibitor methyl-β-cyclodextrin (MβCD, 5 mM, Sigma-Aldrich, USA). After treatment, the cells were harvested in preparation for further experiments.

### CCK-8 assay

Cells were seeded in 96-well plates. After incubating for 24 h, 48 h, and 72 h, the medium was replaced with CCK-8 solution (100 μl, Solarbio, China), and the culture was incubated for 2 h. The sample absorbance (450 nm) was measured by a microplate reader (Biotek Instruments Inc., USA), and each sample was measured three times.

### Colony formation assay

Cells were plated in 6-well plates. After 2 weeks, the colonies were photographed after being fixed in 4% paraformaldehyde and stained with 0.1% crystal violet solution. The colonies were counted using ImageJ software.

### Invasion and migration assay

Cells suspended in serum-free medium were seeded into the top compartment of 24-well transwell plates with an 8 μm pore size (Corning, USA). The top compartments were plated with Matrigel (BD Biosciences, USA) for the invasion assay. Subsequently, 600 μl full medium was added to the bottom compartment. After 24 h, the nonmigrating cells were removed. The cells in the lower chamber were fixed with 4% paraformaldehyde, dyed with 0.1% crystal violet solution, and photographed.

### Flow cytometry

To measure GD2 expression in tumor cells, first, the cells were digested with 0.25% trypsin. Subsequently, a single cell suspension was prepared and incubated with 5 μl of anti-GD2 antibody (Clone 14.G2a, BD Biosciences) for 30 min at 4 °C. After being washed twice, the cells were treated with a fluorescence-labeled secondary antibody for 30 min at 4 °C. To assess the frequency of CD8^+^ T cells in tumor tissues, single-cell suspensions of tumor tissues from 9464D tumor-bearing mice were obtained using the Tumor Dissociation Kit (Miltenyi Biotech, Germany). Cells were treated with FITC-CD3, APC-Cy7-conjugated CD4, and PE-Cy7-conjugated CD8 antibodies (Table S2) for 30 min at 4 °C. Negative control experiments (treatment with the appropriate isotype control Ab) were conducted. The data were collected using a CytoFLEX LX flow cytometer (Beckman, USA) and analyzed by Flow Jo software.

### Immunofluorescence

Cell climbing sheets were prepared, fixed with 4% paraformaldehyde for 10 min, and then blocked with 10% goat serum for 30 min. Next, the cells were treated with anti-GD2 and anti-caveolin-1 overnight at 4 °C (Table S2). Then, the cells were incubated with FITC-conjugated and Cy3-conjugated antibodies for 1 h at room temperature. Negative control experiments were conducted. Following nucleus staining with 4′,6-diamidino2-phenylindole (DAPI) (Solarbio, China), images were obtained using a Zeiss LSM880 confocal microscope (Zeiss, Germany) at 630× magnification. Fluorescence colocalization analysis was performed by ImageJ software.

### Lipid raft extraction

First, the transfected cells were treated with the anti-GD2 antibody ch14.18/CHO (dinutuximab beta, 1000 ng/ml, Rentschler Biopharma SE, Germany), disialoganglioside GD2 (NH4 + salt) (1000 ng/ml, Matreya LLC, USA), and recombinant mouse hepatocyte growth factor (HGF, 40 ng/ml, Solarbio, China). Then, lipid rafts were extracted using the Minute Plasma Membrane-Derived Lipid Raft Isolation Kit (Invent Biotechnologies, USA) according to the manufacturer’s instructions.

### Analysis of secretory CXCL9 and CXCL10

The secretion levels of the chemokines CXCL9 (MIG) and CXCL10 (IP-10) in the cell culture medium were determined using the mouse CXCL9 and CXCL10 ELISA Kit (Biolab, China). The chemokine concentrations were determined by plotting a curve constructed using the recombinant molecule.

### Mouse CD8^+^ T-cell isolation and chemotaxis assay

CD8^+^ T cells were isolated from the spleens of wild type (WT) C57BL/6 mice using the mouse CD8a^+^ T-Cell Isolation Kit (Miltenyi Biotec, Germany). Cells were cultured with RPMI 1640 supplemented with 10% FBS, 1% P/S, and 100 U/ml recombinant murine IL-2. To perform a chemotaxis assay, 24-well Transwell chambers with 5-μm polycarbonate membranes were used. Recombinant murine CXCL9 (100 ng/ml, PeproTech, USA), CXCL10 (50 ng/ml, PeproTech, USA), and culture supernatants from transfected cells after 48 hours of culture were added to the bottom wells. Then, 1 × 10^5^ CD8^+^ T cells were seeded in the top chamber. After incubation for 2 h, the number of migrating cells was assessed using a hemocytometer.

### Quantitative real-time PCR (qRT-PCR)

TRIzol reagent (Invitrogen, USA) was used to obtain total RNA, and PrimScript RT Master Mix was used to perform reverse transcription (Takara, Japan). Then, qRT-PCR was performed using the SYBR Green PCR Kit (Takara, Japan) in an ABI QuantStudio 5 (Q5) system (Applied Biosystems, USA). The cycling characteristics were as described below: 95 °C/5 s and 60 °C/34 s for 40 cycles. The mRNA level was normalized to GAPDH. The relative expression level was determined using 2^−ΔΔCt^. All reactions were performed in triplicate. The primers for the target genes are listed in Table S4.

### Western blot analysis

RIPA lysis buffer (Solarbio, China) was used to harvest total protein. The BCA approach (Thermo, USA) was used to determine the protein concentration. Protein samples were separated using SDS-PAGE gels and transferred to PVDF membranes. After blocking with 5% skimmed milk powder for 1 h, the membranes were treated with the primary antibodies at 4 °C overnight. The primary antibodies are listed in Table S2. The membranes were treated with the secondary antibodies for 1 h at room temperature. The band images were created by a GelView 6000Plus system (Biolight Biotechnology, China) and quantified by ImageJ software. β-actin was used as an internal control.

### Establishment of a xenograft model

All animal experiments were carried out following the Guide for the Care and Use of Laboratory Animals and were authorized by the Institutional Animal Care and Use Committee of the Tianjin Medical University Cancer Institute and Hospital (AE-2021112). Female C57BL/6 mice (6–8 weeks, weighing 18–20 g) were purchased from Beijing Vital River Laboratory Animal Technology Co. Ltd. (Beijing, China) and housed under specific pathogen-free conditions. In each treatment group, 5 mice were used to investigate changes in the tumor phenotype.

To examine the inhibitory effects of B3GALT4 on tumor growth in vivo, the vector and B3GALT4-overexpressing 9464D cells (2 × 10^6^/100 μl PBS per mouse) were injected into the backs of the mice. To further confirm whether a lipid raft inhibitor can reverse the knockdown of B3GALT4-mediated immunosuppressive effects in vivo, sh-B3GALT4 9464D cells (2 × 10^6^/100 μl PBS per mouse) were injected into the backs of the mice. On Day 6 after tumor implantation, mice were treated with the lipid raft inhibitor MβCD (300 mg/kg) or PBS every 3days for 18 days. To assess the antitumor effect of MβCD combined with anti-GD2 mAb in NB xenograft models, 9464D cells (2 × 10^6^/100 μl PBS per mouse) were injected into the backs of the mice. On Day 6 after tumor implantation, MβCD was injected intraperitoneally (i.p.) (300 mg/kg) every 3 days, and anti-GD2 mAb (200 μg/per mouse, i.p.) was injected twice weekly for 18 days. According to the algorithm, the tumor volume was analyzed every 3 days: (length × width^2^)/2. After the mice were euthanized, the tumors were separated, weighed, and harvested for subsequent analysis.

### Statistical analysis

Data analysis and graphing were conducted with Prism 8.0 software (Graph Pad Software Inc.). Quantitative data are presented as the mean ± standard deviation. An unpaired Student’s t test was used to analyze differences between the two groups. Statistically significant differences were analyzed using one-way ANOVA (Bonferroni test) among multiple groups. A *P* value of less than 0.05 was regarded as statistically significant, and “ns” indicated “not significant”. The *P* values in the figures are depicted with the following symbols: ^*^ *P* < 0.05, ^**^ *P* < 0.01, ^***^ *P* < 0.001, ^****^ *P* < 0.0001.

## Results

### GD2 is overexpressed in NB tissues and mediated by B3GALT4

**Fig. 1:**
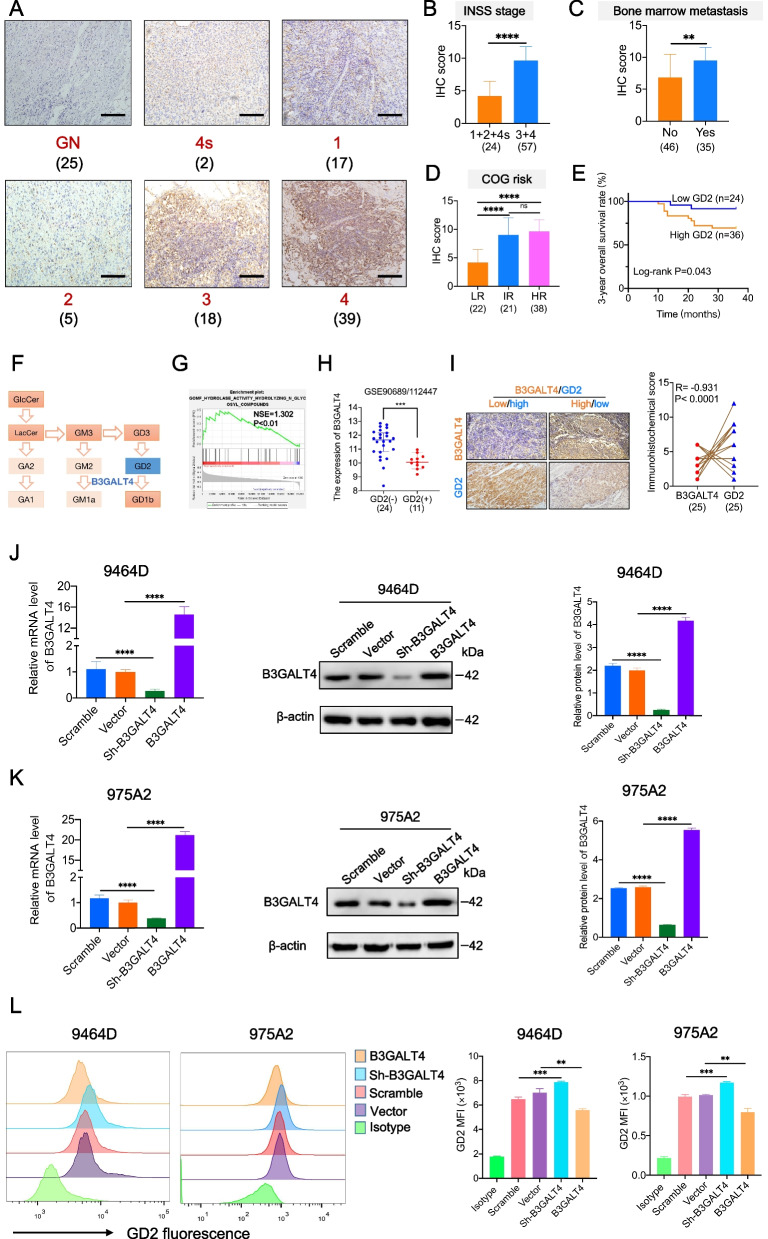
Overexpression of GD2 in NB tumor tissues is mediated by B3GALT4. **A** Immunohistochemistry (IHC) analysis of GD2 expression in ganglioneuroma (GN) (*n* = 25) and neuroblastoma (NB) tumor tissues (*n* = 81). Scale bar: 100 μm. **B** The correlation between the International Neuroblastoma Staging System (INSS) stage and GD2 expression of NB was analyzed. **C** IHC scores were applied to assess GD2 expression in NB specimens with or without bone marrow metastasis. **D** The correlation between the Children’s Oncology Group (COG) stage and GD2 expression of NB was analyzed. **E** Kaplan-Meier survival curve for 3-year overall survival of 60 NB patients in the 81 patients (Fig. 1A-D) according to GD2 expression levels (log-rank test and the *p*-value is shown). **F** B3GALT4 was a crucial enzyme for ganglioside GD2 synthesis. **G** Gene set enrichment analysis (GSEA) analysis showed that B3GALT4 overexpression was enriched in hydrolyzing N-glycosyl compounds based on data from the GEO database (GSE49710). **H** Differences in B3GALT4 level between GD2 (+) and GD2 (−) NB samples from the GEO databases (GSE90689 and GSE112447). **I** Negative correlation between the expression level of GD2 and B3GALT4 in NB tissues (n = 25). Scale bar: 100 μm. **J**, **K** B3GALT4 knockdown and overexpression 9464D and 975A2 stable cell lines were constructed using recombinant lentiviral transfection. The overexpression and knockdown efficiency were detected by quantitative real-time polymerase chain reaction (qRT-PCR) and western blotting. Quantitative data are shown as means ± SEM from three independent experiments. Representative Western blots and bar charts are shown. (L) Representative flow cytometric analysis images of GD2 levels in 9464D and 975A2 cells with different treatments and statistical analysis results

Disialoganglioside GD2 is an important target for immunotherapy of NB. Here, we confirmed that GD2 was overexpressed in NB tissues compared with GN tissues by IHC (Fig. 1A). In addition, high GD2 expression was related to advanced INSS stage, bone marrow metastasis, and COG high-risk classification (Fig. 1B-D). Kaplan-Meier analysis results showed that a higher GD2 level was correlated with a shorter 3-year overall survival (OS) of NB patients (Fig. 1E).

As shown in Fig. 1F, B3GALT4 is a crucial glycosyltransferase involved in synthesizing ganglioside GD2. The GSEA results also demonstrated that B3GALT4 was mainly enriched in hydrolyzing N-glycosyl compounds involved in ganglioside GD2 metabolism (Fig. 1G). According to the analysis of the GEO databases, B3GALT4 expression was significantly reduced in GD2 (+) NB tissues compared with GD2 (−) NB tissues (*P* < 0.001) (Fig. 1H). We subsequently evaluated GD2 and B3GALT4 protein expression levels in human NB clinical samples by IHC. We also observed a strong negative connection between the levels of GD2 and B3GALT4 (*r* = − 0.931, *P* < 0.0001) (Fig. 1I). To further evaluate whether the GD2 expression level was mediated by B3GALT4, we established stable NB cell lines with B3GALT4 knockdown (sh-B3GALT4) and B3GALT4 overexpression (B3GALT4) NB cell lines (Fig. 1J and K) and analyzed the expression level of GD2 in these cells using a flow cytometry assay. We found that B3GALT4 knockdown significantly upregulated GD2 expression levels in the NB cell lines 9464D and 975A2, while B3GALT4 overexpression significantly downregulated GD2 levels (Fig. 1L). The above data indicated that GD2 was overexpressed in NB tissues and was mediated by B3GALT4.

### B3GALT4 is downregulated in NB tissues, and deficient B3GALT4 suggests an unfavorable prognosis

**Fig. 2:**
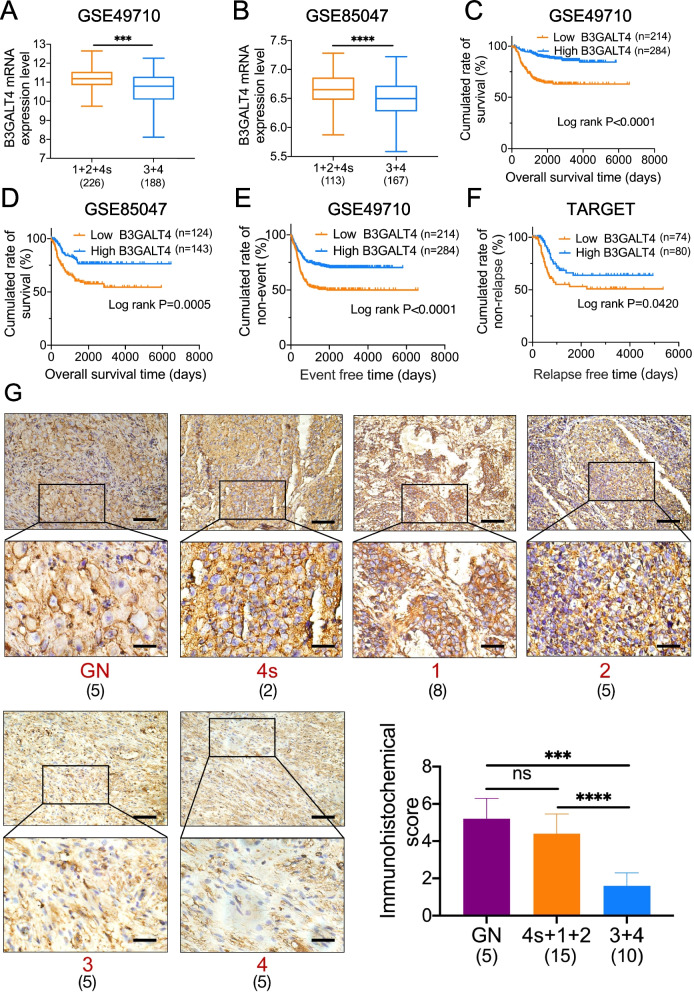
B3GALT4 is down-regulated in NB tissues, and deficient B3GALT4 indicates a poor prognosis. A bioinformatics study was conducted to analyze the B3GALT4 expression levels in INSS stages 1 + 2 + 4 s versus 3 + 4 NB patients based on the GSE49710 (**A**) and GSE85047 (**B**) datasets. Kaplan-Meier analysis of overall survival (OS) (**C, D**), event-free survival (EFS) (**E**), and relapse-free survival (RFS) (**F**) using the TARGET database and GEO datasets median expression value cutoffs for B3GALT4. *P*-value was determined by a log-rank test. **G** IHC was performed to detect B3GALT4 expression in GN (*n* = 5) and NB (n = 25) tumor tissues (scale bars of the initial images: 100 μm, inset: scale bars of magnification: 25 μm)

Given the role of B3GALT4 in regulating ganglioside GD2 synthesis, we explored the association between B3GALT4 expression levels and the progression of NB. NB data from two GEO datasets (GSE49710 and GSE85047) showed that low expression of B3GALT4 was associated with advanced INSS stage (Fig. 2A and B). NB patients with low B3GALT4 expression had shorter OS (Fig. 2C and D), event-free survival (EFS) (Fig. 2E), and relapse-free survival (RFS) (Fig. 2F) than patients with elevated expression. We then analyzed the expression of B3GALT4 in NB samples from patients with different clinical stages by IHC. The results showed that B3GALT4 IHC scores in advanced stages of NB tissues were significantly lower than those in early-stage tissues (*P* < 0.0001) (Fig. 2G). The above findings suggested that B3GALT4 is a potential prognostic biomarker in patients with NB.

### Elevated B3GALT4 inhibits NB progression in vitro and in vivo

**Fig. 3:**
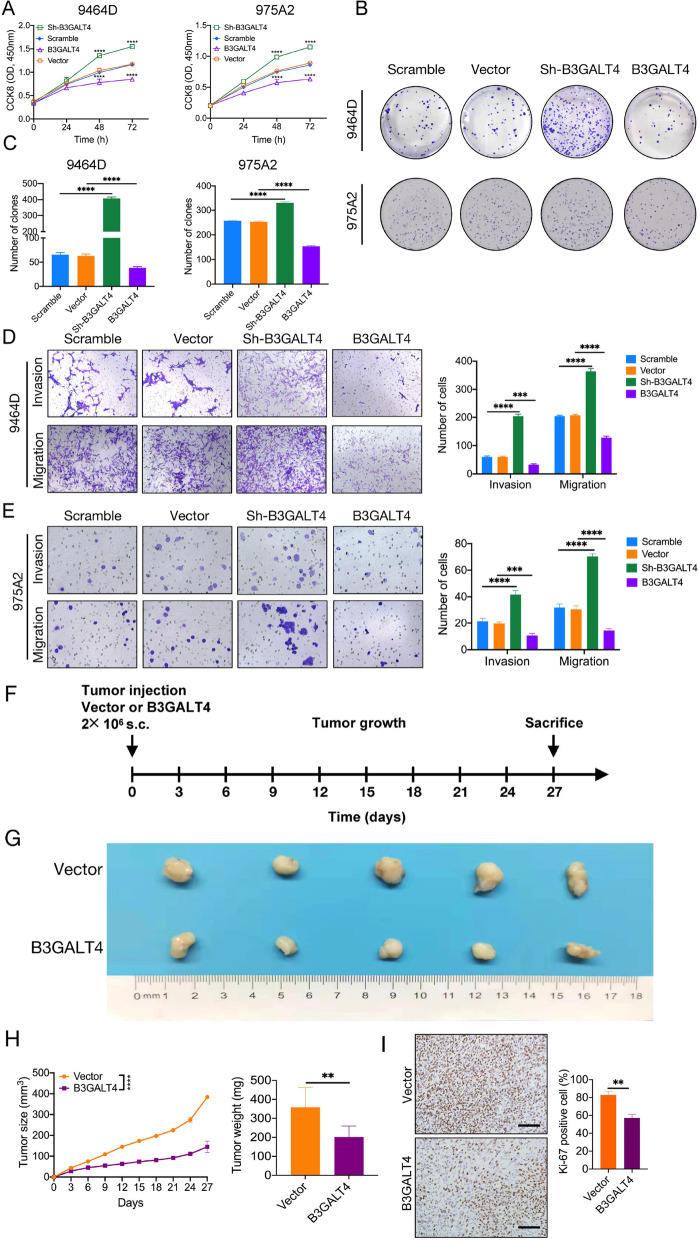
Elevated B3GALT4 inhibits NB progression in vitro and in vivo. CCK-8 (**A**) and colony formation assays (**B, C**) were performed to determine the proliferation of 9464D and 975A2 cells transfected with B3GALT3 overexpression and knockdown lentivirus. Transwell assays were performed to analyze the invasion and migration abilities of 9464D (**D**) and 975A2 (**E**) cells. **F** Approximately 2 × 1069464D cells infected with vector or overexpressed B3GALT4 plasmids were injected subcutaneously into the back of C57BL/6 mice (n = 5 mice per group). **G** After 27 days, the mice were sacrificed, and the tumors were harvested. **H** The tumor volumes were measured every 3 days and shown in the line chart (Left). The final weights of tumors were calculated (Right). **I** IHC staining for Ki-67 in the indicated tumors. Scale bar: 100 μm

We then investigated the biological function of B3GALT4 in NB cells. The results showed that overexpression of B3GALT4 decreased the proliferation, migration, and invasion of 9464D and 975A2 cells. In contrast, knockdown of B3GALT4 in the 9464D and 975A2 cell lines enhanced proliferation, migration, and invasion (Fig. 3A-E).

To better evaluate the effect of B3GALT4 on NB growth in vivo, we established a subcutaneous model with B3GALT4-overexpressing 9464D cells in C57BL/6 mice (Fig. 3F). The results showed that upregulation of B3GALT4 dramatically inhibited tumorigenesis (Fig. 3G and H). In addition, IHC staining of Ki-67 revealed that the number of the Ki-67-positive cells in tumors of the B3GALT4 group were lower than those in the vector group (*P* < 0.01) (Fig. 3I), suggesting that B3GALT4 overexpression inhibited the proliferation of 9464D tumors. These data demonstrated that B3GALT4 serves as a tumor suppressor gene in NB.

### Elevated B3GALT4 induces CD8^+^ T-cell infiltration mediated by CXCL9 and CXCL10

**Fig. 4:**
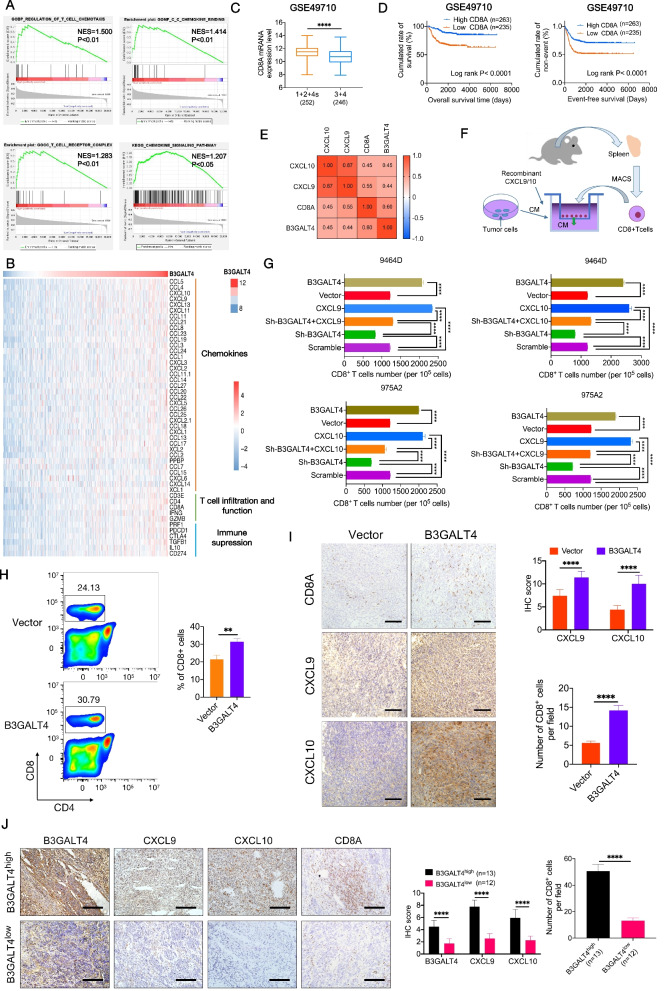
B3GALT4 modulates CD8+ T lymphocyte migration and tumor infiltration via CXCL9 and CXCL10. **A** GSEA analysis indicated that B3GALT4 overexpression was enriched in T cell chemotaxis and chemokine-mediated signaling pathways based on data from the GEO database (GSE49710). **B** Integrated molecular analysis of mRNA expression of genes from the GSE49710 dataset was performed based on the B3GALT4 mRNA expression level. **C** Box plot demonstrating CD8A mRNA expression level in NB tissues (stages 1 + 2 + 4 s versus stages 3 + 4) from the GSE49710 dataset. **D** Survival analysis showed CD8A expression levels were significantly related to EFS and OS of NB patients in the GSE49710 dataset. **E** Correlation analysis between B3GALT4, CD8A, and chemokines (CXCL9 and CXCL10) mRNA expression levels in the GSE49710 database. **F** Schematic diagram of in vivo chemotaxis assay of CD8+ T cell migration. **G** Transwell assay analysis of CD8+ T cell migration ability in the supernatants of tumor cells with different treatments. **H** The percentages of CD8+ T cells in tumors from subcutaneous xenograft mouse models were assessed by FACS. Bar graphs showed the mean ± SD of three independent experiments. **I** IHC was done to evaluate CD8, CXCL9, and CXCL10 expression in NB tissues derived from tumor xenografts in mice. **J** IHC staining results of the B3GALT4, CD8A, CXCL9, and CXCL10 expression in tumor samples from NB patients with B3GALT4 high (*n* = 13) or low expression (*n* = 12)

To assess whether the antitumor activity of B3GALT4 overexpression was related to modulation of the TME, we first conducted GSEA based on data from the GSE49710 dataset. In comparison to the B3GALT4 low expression group, the high expression group was primarily enriched in the regulation of T-cell chemotaxis, T-cell receptor complex, C-C chemokine binding, and the chemokine signaling pathway (Fig. 4A). Increasing evidence indicates that aberrant glycosylation of cancer cells plays a key role in the modulation of the TME [23–25]. Previously, we found that the glycosyltransferase signature was related to CD8^+^ T-cells infiltration of NB [26]. However, the correlation between B3GALT4 expression and T-cell infiltration is still unknown in NB. Therefore, we investigated the correlations between B3GALT4 expression levels and known human chemokines, T-cell infiltration and function, and immunosuppression based on the GSE49710 dataset. The results indicated that higher B3GALT4 expression was related to elevated expression levels of T-cell-related chemokines (CXCL9 and CXCL10) and T-cell markers (CD3, CD4, and CD8A) (Fig. 4A). Several studies verified the relationship between enhanced T lymphocyte infiltration and better outcomes in patients with NB [27–29]. Given the roles of CD8^+^ T cells in NB outcome, we analyzed the relationship between CD8A level and tumor stages and the prognosis of NB patients from the GSE49710 dataset. As shown in Fig. 4C, decreased expression of CD8A was observed in advanced stages of NB. High CD8A expression was associated with longer OS (*P* < 0.0001) and EFS (*P* < 0.0001) (Fig. 4D). Moreover, the expression level of B3GALT4 was significantly positively correlated with CD8A, CXCL9, and CXCL10 levels in clinical NB samples from the GSE49710 dataset (Fig. 4E). Recently, CXCL9 and CXCL10 were reported to be necessary for the trafficking of activated CD8^+^ T cells into tumor sites [30–32]. To explore whether B3GALT4 mediated CD8^+^ T-cell infiltration via CXCL9 and CXCL10, we conducted a migration assay in vitro. First, CD8^+^ T cells were isolated from the spleens of WT C57BL/6 mice. Then, a chemotaxis assay was carried out (Fig. 4F). The results demonstrated that the supernatant of B3GALT4-overexpressing tumor cells markedly promoted the migration of CD8^+^ T cells. However, the supernatant of sh-B3GALT4-transfected NB cells strongly inhibited the migration of CD8^+^ T cells (Fig. 4G). Furthermore, recombinant murine CXCL9 or CXCL10 alone significantly induced CD8^+^ T-cell migration (Fig. 4G). A rescue study was performed to observe the effect of the chemokines CXCL9 and CXCL10 on B3GALT4 inhibition-induced CD8^+^ T-cell recruitment. Sh-B3GALT4-transfected 9464D and 975A2 cellular supernatants were treated with the chemokines CXCL9 and CXCL10, respectively. The results showed that concurrent treatment with chemokines induced sh-B3GALT4-inhibited CD8^+^ T-cell migration in both cell lines (Fig. 4G). To confirm the impact of upregulation of B3GALT4 on CD8^+^ T-cell migration in vivo, flow cytometry was used to determine the number of CD8^+^ T cells in tumors from subcutaneous xenograft mouse models. The results demonstrated that the CD8^+^ T cells were accumulated more in the stroma of B3GALT4-overexpressing tumors than in the stroma of vector-treated tumors (Fig. 4H). We further confirmed that the expression levels of CXCL9, CXCL10, and CD8A in the B3GALT4 overexpression group were higher than those in the vector group by an IHC assay (Fig. 4I). In addition, B3GALT4 expression was positively correlated with CXCL9/CXCL10 expression and CD8^+^ T-cell infiltration in human NB clinical samples (Fig. 4J). Accordingly, these data provide strong evidence that B3GALT4 overexpression may promote CD8^+^ T lymphocyte recruitment into tumor sites via CXCL9 and CXCL10 in NB.

### B3GALT4 regulates lipid raft formation via GD2

**Fig. 5:**
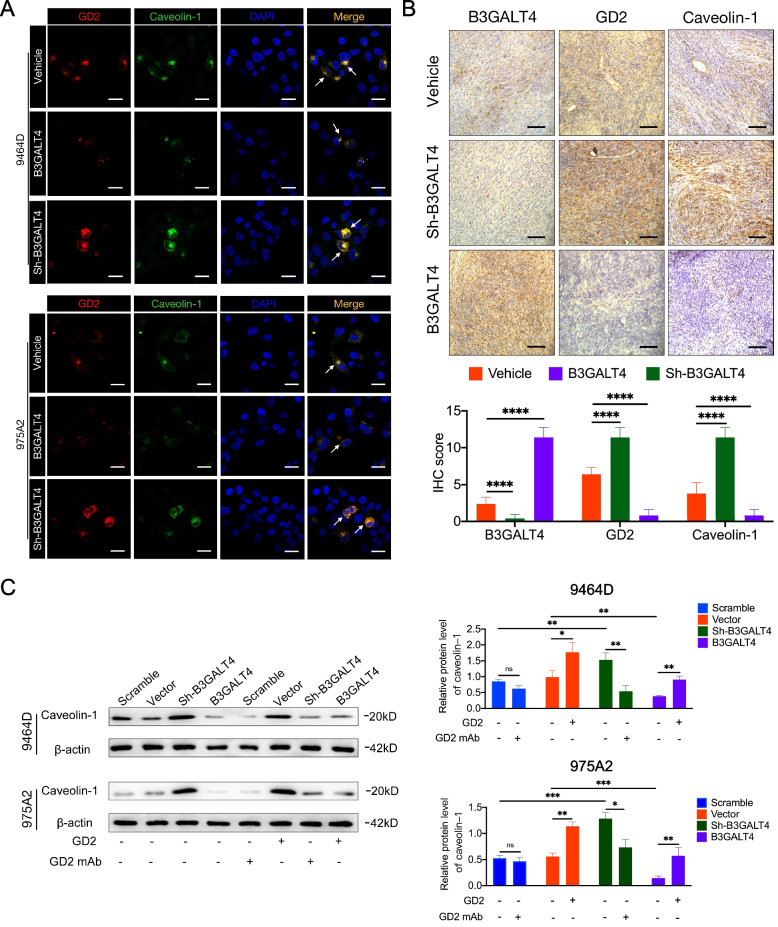
Modulatory role of ganglioside GD2 on lipid rafts. **A** Immunofluorescence colocalization of GD2 and caveolin-1 in 9464D and 975A2 cells with silenced or overexpression of B3GALT4. Scale bar: 50 μm. **B** IHC was used to evaluate B3GALT4, GD2, and caveolin-1 expression in NB tissues derived from tumor xenografts in mice, *n* = 5. Scale bar: 100 μm. **C** Western blot was conducted to analyze the expression of caveolin-1 in lipid rafts of 9464D and 975A2 cells with treatment with ganglioside GD2 or GD2 mAb

Studies have shown that lipid rafts are essential in regulating tumor progression [33–35]. Gangliosides are mainly localized in lipid rafts, and gangliosides GM1 and GD3 modulate lipid raft activity [36, 37]. We investigated whether ganglioside GD2 could similarly regulate lipid raft formation. As caveolin-1 is a lipid raft marker, we first explored whether GD2 was colocalized with caveolin-1 by double immunofluorescence staining. The results demonstrated that GD2 was colocalized with caveolin-1 on the plasma membrane of NB cells (Fig. 5A). Then, we further analyzed the expression levels of GD2 and caveolin-1 by IHC in tumors from subcutaneous xenograft mouse models. The results confirmed that the expression level of caveolin-1 was decreased in the B3GALT4 overexpression group (low GD2 expression) and upregulated in the B3GALT4 low expression group (high GD2 expression) compared with the vehicle group (Fig. 5B). To further investigate the role of ganglioside GD2 mediated by B3GALT4 in lipid raft formation, we extracted lipid rafts and evaluated the expression of caveolin-1 in NB cells with different treatments. First, purified ganglioside GD2 alone significantly enhanced caveolin-1 protein levels in lipid raft fractions (Fig. 5C). Caveolin-1 expression levels were significantly decreased in B3GALT4-overpressing NB cells compared with vector-treated cells. However, the protein expression levels of caveolin-1 were significantly increased in B3GALT4-overpressing cells after purified ganglioside GD2 treatment. Meanwhile, the protein expression levels of caveolin-1 were increased by B3GALT4 silencing, whereas the addition of anti-GD2 mAb reversed this effect (Fig. 5C). These results showed that GD2 mediated by B3GALT4 played an essential role in lipid raft formation.

### B3GALT4 regulates CXCL9 and CXCL10 expression through c-met signaling in the lipid rafts and the downstream AKT/mTOR/IRF-1 pathway

**Fig. 6:**
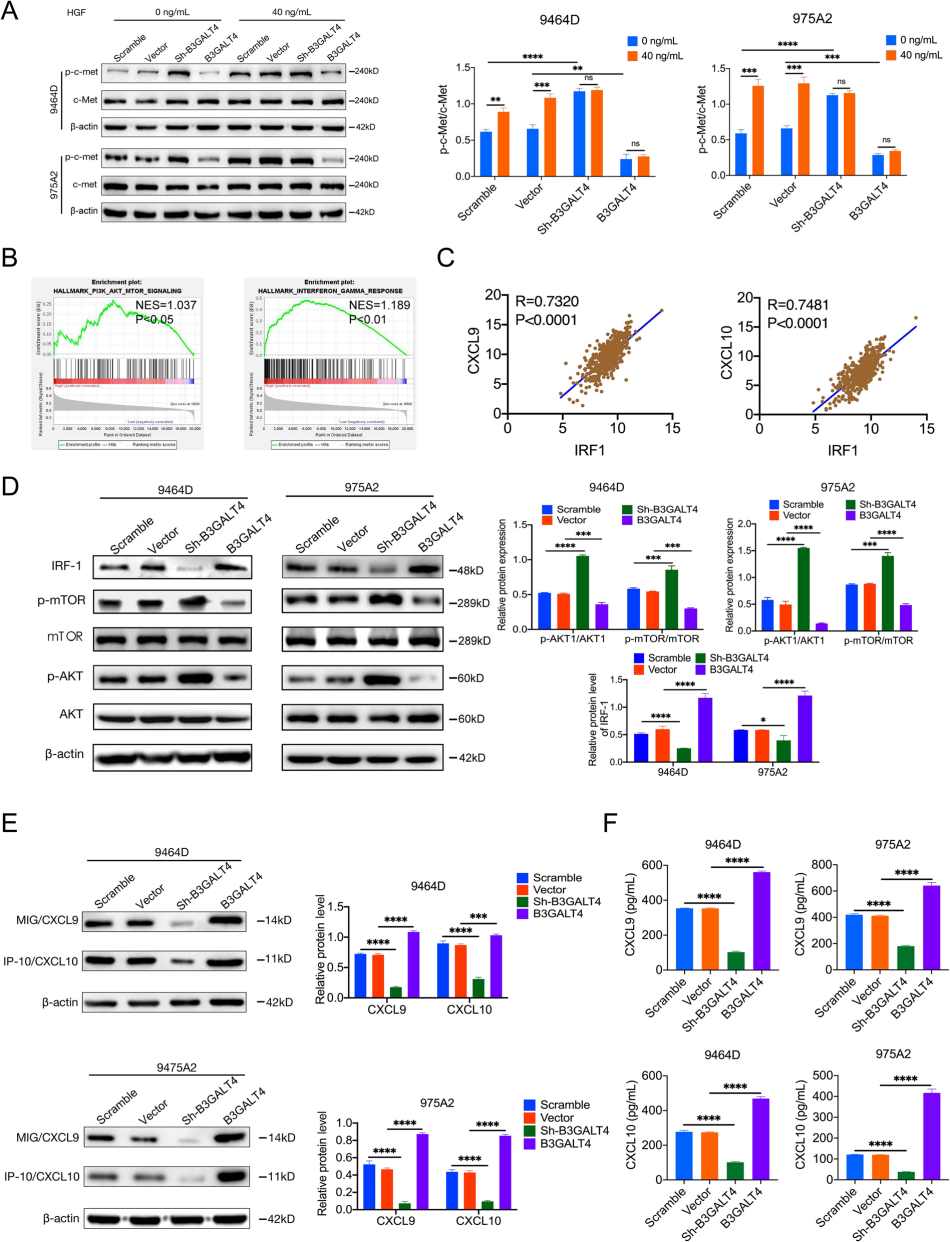
The c-Met/AKT/mTOR/IRF-1 pathway is essential for synthesizing CXCL9 and CXCL10 regulated by B3GALT4. **A** Western blot analysis of the protein level of c-Met and the phosphorylation of c-Met in lipid rafts of 9464D and 975A2 cells with treatment with hepatocyte growth factor (HGF) (40 ng/ml, 24 h). **B** GSEA analysis showed that B3GALT4 overexpression was enriched in PI3K/AKT/mTOR and interferon regulatory factor-1 (IFN-γ) pathways based on data from the GEO database (GSE49710). (**C**) Correlation analysis of CXCL9, CXCL10, and IRF-1 expression levels in human NB specimens from the GSE49710 database. **D**, **E** Western blot analysis of the protein level of mTOR, p-mTOR, AKT, p-AKT, IRF-1, CXCL9, and CXCL10 in 9464D and 975A2 cells. **F** Enzyme-linked immunosorbent assay (ELISA) analysis of CXCL9 and CXCL10 in the 9464D and 975A2 cells supernatants

We next explored how upregulation of B3GALT4 induced CXCL9/CXCL10 expression. The HGF/c-Met pathway in lipid rafts plays a significant role in the tumor microenvironment [38, 39]. It has recently been reported that the clustering of GD2 activates c-Met [40]. Based on previous studies, we sought to determine whether B3GALT4 regulates CXCL9 and CXCL10 production through c-Met signaling in lipid rafts. We first extracted lipid rafts and observed the phosphorylated c-Met (p-c-Met) levels by western blot. The results showed that the expression levels of p-c-Met in lipid rafts were significantly increased in B3GALT4-knockdown cells and decreased in B3GALT4-overexpressing cells compared with the vehicle cells (Fig. 6A), indicating that B3GALT4 downregulation activated the c-Met signaling pathway in lipid rafts (Fig. 6A). Since the activation of c-Met depends on HGF, we next investigated whether HGF could affect the activation of c-Met mediated by B3GALT4 downregulation. Western blot analysis showed that HGF treatment increased p-c-Met levels in the vehicle cells, but no changes in the levels of p-c-Met were observed in B3GALT4-knockdown or B3GALT4-overexpressing cells after HGF treatment (Fig. 6A). These results indicate that B3GALT4 downregulation can activate c-Met independent of HGF. GSEA was conducted to explore the downstream signaling pathway based on data from the GSE49710 dataset. The results showed that the PI3K/AKT/mTOR and IFN-γ signaling pathways were significantly enriched in samples with high B3GALT4 expression (Fig. 6B). It is well established that the IFN-γ signaling pathway regulates CXCL9 and CXCL10 expression in cancer cells [41]. IRF-1 is an IFN-γ/p-STAT1 responsive transcription factor that governs immune-related genes, such as genes of the CXCL and CCL families [42]. We found that the expression of IRF-1 was significantly positively correlated with the expression of CXCL9 and CXCL10 in NB tissues from the NB dataset (GSE49710) (Fig. 6C). Accordingly, western blot analysis showed that the level of IRF-1 was dramatically decreased in B3GALT4-knockdown cells and increased in B3GALT4-upregulated cells (Fig. 6D). A previous study showed that the inhibition of the PI3K/AKT/mTOR signaling pathways elevates the expression of the chemokines CXCL10 and CXCL11 expression via IRF-1 [19]. In our study, western blot analysis revealed that the expression levels of p-AKT and p-mTOR were significantly increased in B3GALT4-knockdown cells and significantly decreased in B3GALT4-overexpression cells (Fig. 6D). Moreover, the results showed that overexpression of B3GALT4 enhanced CXCL9 and CXCL10 synthesis, but downregulation of B3GALT4 reduced the levels of these two chemokines, as shown by western blotting (Fig. 6E) and the ELISA test (Fig. 6F). These findings confirmed the conclusion that the c-Met/AKT/mTOR/IRF-1 pathway mediated by B3GALT4 is involved in CXCL9 and CXCL10 production.

### The lipid raft inhibitor MβCD suppresses the knockdown of B3GALT4-mediated tumor progression and immune escape

**Fig. 7:**
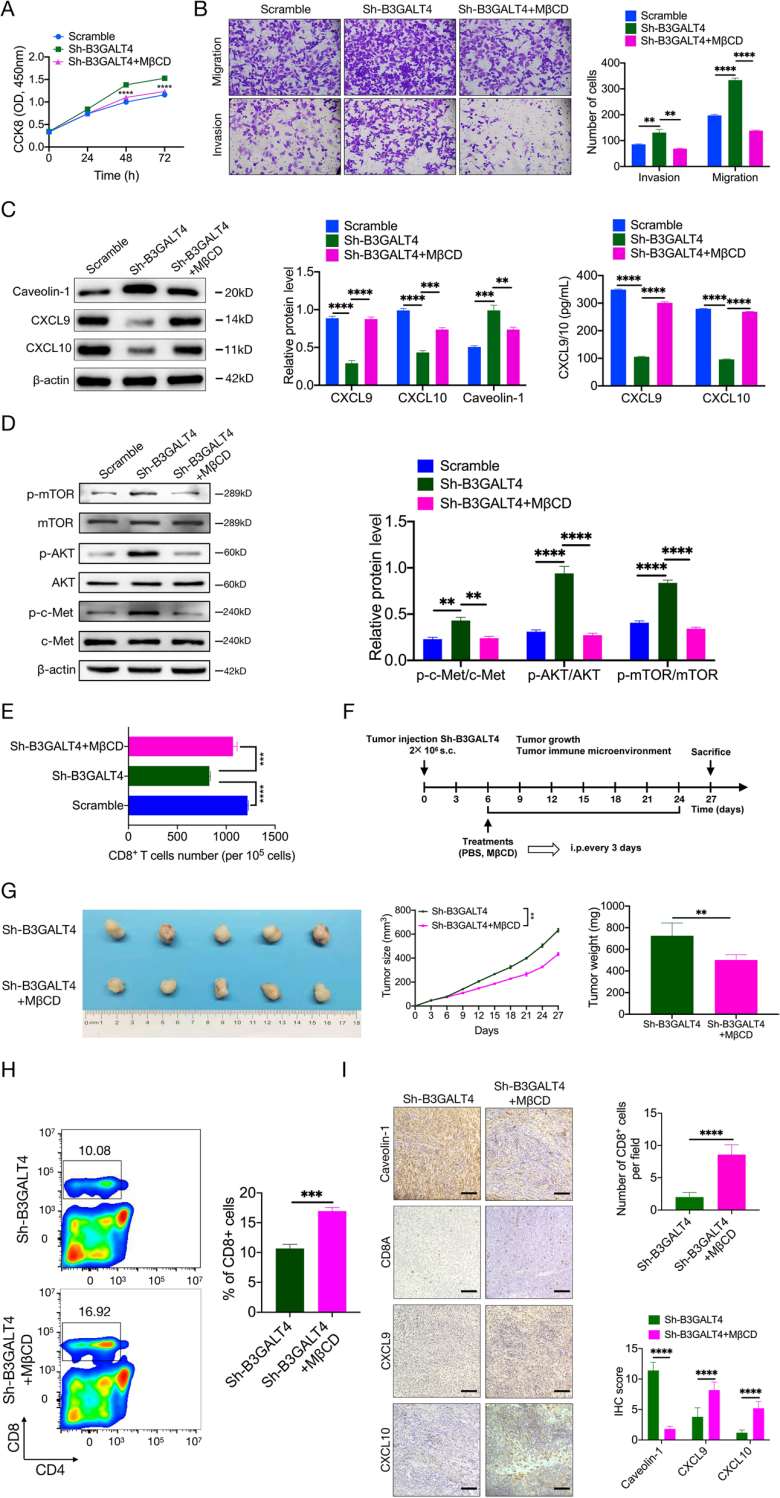
Treatment of lipid raft inhibitor, MβCD, suppresses low B3GALT4-mediated NB progression and CD8+ T cells recruitment. **A** The effects of MβCD on 9464D cell proliferation were measured by a CCK-8 assay. **B** The impacts of MβCD on 9464D cell migration and invasion. **C** Western blot and ELISA have shown that the treatment of MβCD can enhance knockdown B3GALT4-mediated CXCL9 and CXCL10 production and inhibit caveolin-1 expression in lipid rafts. **D** Western blot showed that the treatment of MβCD could reverse knockdown B3GALT4-mediated activation of the c-Met/AKT/mTOR pathway. **E** Chemotaxis assays showed that the treatment of MβCD could improve knockdown B3GALT4-mediated CD8+ T cell migration. **F** The in vivo experimental procedure scheme was detailed in the “Materials and Methods” and “Results” sections. **G** In vivo assays showed that the treatment of MβCD could block knockdown B3GALT4-mediated NB growth. **H** Flow cytometric analyzed the population of intratumoral CD8+ T cells between groups on day 27, respectively, n = 5. **I** Representative IHC images of serial sections derived from the tumor xenografts were stained for caveolin-1, CD8A, CXCL9, and CXCL10. Left, representative pictures of IHC staining (scale bar: 100 μm). Right, a statistic of CD8+ T cells per section, the digital quantification of the histoscore of CXCL9 and CXCL10

To further verify whether B3GALT4 downregulation promoted NB progression through lipid rafts, rescue experiments were carried out. MβCD can remove cellular cholesterol and is commonly used as a lipid raft inhibitor [43]. As observed previously, B3GALT4-knockdown 9464D cells exhibited higher proliferation, invasion, and migration abilities than untreated 9464D cells. However, this difference was abolished by MβCD treatment (Fig. 7A and B). The expression levels of caveolin-1, CXCL9, and CXCL10 were significantly increased after MβCD treatment compared with the B3GALT4 knockdown group (Fig. 7C). We next demonstrated that MβCD reversed the effect of B3GALT4 knockdown on the c-Met/AKT/mTOR pathway (Fig. 7D). Meanwhile, MβCD markedly reversed the inhibitory effect of B3GALT4 knockdown on the migration of CD8^+^ T cells (Fig. 7E). Subcutaneous tumor models with B3GALT4-knockdown 9464D cells confirmed that MβCD relieved the knockdown of B3GALT4-mediated tumor growth (Fig. 7F and G). In accordance with the chemotaxis assay data, MβCD treatment enhanced the number of CD8^+^ T cells in tumor tissues of C57BL/6 mice treated with B3GALT4-knockdown 9464D cells (Fig. 7H). IHC staining of tumor tissues from mice treated with B3GALT4 knockdown 9464D cells confirmed the expression levels of CXCL9 and CXCL10, and the percentage of CD8A-positive cells was increased after MβCD treatment, while caveolin-1 was decreased (Fig. 7I). Taken together, these data demonstrated that MβCD attenuated the knockdown of B3GALT4-induced tumor progression and immunosuppression.

### Combination therapy of anti-GD2 immunotherapy with MβCD promotes CD8^+^ T-cell infiltration via CXCL9 and CXCL10.

Anti-GD2 mAbs have shown certain clinical efficacy for patients with high-risk NB [44]. Unfortunately, some NB patients do not respond to anti-GD2 mAb treatment due to various immunosuppressive factors, such as a deficiency of CD8^+^ T-cell infiltration [45]. Based on the previous findings, we investigated whether the inhibition of lipid rafts could improve the efficacy of anti-GD2 therapy by examining the antitumor effect of the anti-GD2 mAb in combination with MβCD using a 9464D subcutaneous xenograft tumor mouse model. After xenografts were established, PBS, anti-GD2 mAb, MβCD, or the combination of anti-GD2 mAb and MβCD was administered to the mice (Fig. S1A). The results showed that anti-GD2 mAb combined with MβCD therapy significantly suppressed tumor growth compared with PBS treatment or treatment with MβCD (Fig. S1B-D). The combination of anti-GD2 mAb and MβCD therapy showed only a nonsignificant trend for tumor growth delay compared with anti-GD2 mAb treatment alone. Meanwhile, no statistically significant variations in the average body weight of mice were identified (Fig. S2).

**Fig. 8:**
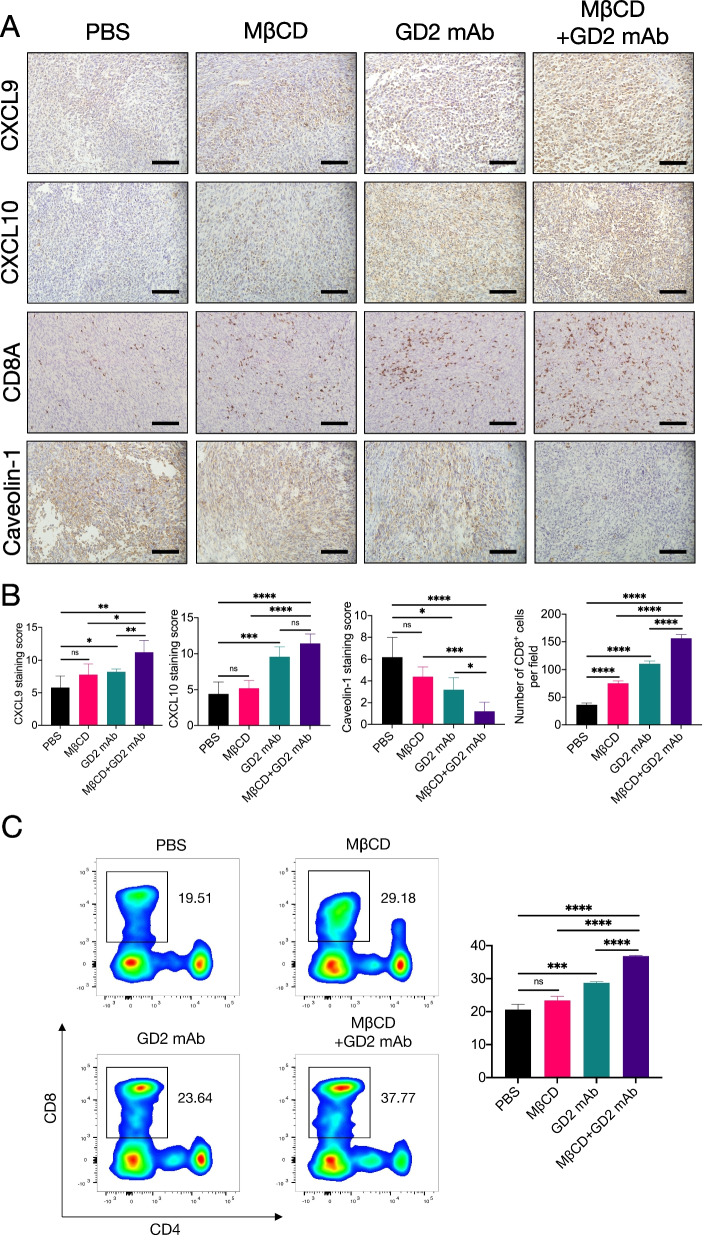
Increased intratumoral CD8+ T cell infiltration after MβCD plus anti-GD2 treatment is mediated by CXCL9 and CXCL10. **A** Representative images of CD8A, CXCL9, CXCL10, and caveolin-1 immunohistochemical staining of tumors treated with PBS, MβCD, or anti-GD2 mAb (scale bar: 100 μm). **B** A bar graph showed the IHC score quantification of CXCL9, CXCL10, and caveolin-1. Quantifications of CD8+ T cells percentage from mice treated with different conditions as indicated. **C** Flow cytometric analysis (left) and statistical analysis (right) of the numbers of CD8+ T cells per mg of tumors in mice 21 days after the beginning of treatments

To further explore the effect of anti-GD2 mAb plus MβCD on tumor immunity, we detected the protein expression of CD8A, caveolin-1, CXCL9, and CXCL10 by IHC staining. We found that the protein levels of CXCL9 and CXCL10 were obviously elevated, and the protein level of caveolin-1 was downregulated in the tumor cells of mice treated with anti-GD2 mAb plus MβCD compared with the other three groups (Fig. 8A and B). Next, we found a higher percentage of CD8A-positive cells in the tumor tissues of mice given anti-GD2 mAb plus MβCD than that in the other three groups (Fig. 8A and B). Furthermore, flow cytometry analysis also showed that the percentage of CD8^+^ T cells was higher in tumor tissues of mice treated with anti-GD2 mAb plus MβCD than in the other three groups (Fig. 8C).

## Discussion

It is becoming more obvious that the role of the tumor microenvironment in the immunotherapy response is extremely important [46–48]. Tumor cells employ inherent controllers to build an immunosuppressive microenvironment to evade T-cell-mediated immune surveillance [49, 50]. Therefore, identifying the genes responsible for immune evasion has been a significant focus of research.

B3GALT4 belongs to the β­1,3­galactosyltransferase gene family, which transfers a galactose to the N-acetylgalactosamine residue of GM2/GD2 with a β1–3 linkage. Studies have demonstrated that B3GALT4 is associated with the prognosis of both ovarian cancer and colorectal cancer [9, 51]. In addition, B3GALT4 overexpression inhibits NB-cell tumorigenesis [11]. However, the relationship between B3GALT4 and the NB immune microenvironment is not entirely understood. In the present study, we demonstrated that B3GALT4 was downregulated in NB tissues and that a deficient B3GALT4 level indicated a poor prognosis. Loss of B3GALT4 promoted NB progression in vitro and in vivo. Furthermore, B3GALT4 mediated lipid raft formation via ganglioside GD2 and CD8^+^ T-cell infiltration triggered by the chemokines CXCL9 and CXCL10. Finally, the lipid raft inhibitor combined with anti-GD2 mAb treatment significantly enhanced the antitumor effect and the infiltration of CD8^+^ T cells.

CD8^+^ T lymphocytes are the main antitumor effector cells. The relationship between enhanced T-cell infiltration and better immunotherapeutic effectiveness in NB has prompted much research of involving immunostimulatory mechanisms to promote T-cell infiltration. Chemokines are widely established to have an important function in the recruitment of immune cells [52]. CXCL9, a monokine induced by gamma interferon (MIG), is induced by IFN-γ [53]. CXCL10, known as interferon γ-induced protein 10 (IP-10), is induced by IFN-γ as well as by IFN-α/β [54]. Previous studies demonstrated that tumor cell-derived CXCL9/CXCL10 modulated T-cell recruitment in various tumors [55, 56]. In our studies, we showed that B3GALT4 overexpression drove CD8^+^ T-cells recruitment by upregulating the chemokines CXCL9 and CXCL10. Therefore, it may be possible to reverse the status of the immune desert by targeting B3GALT4 in NB cells.

Lipid rafts, plasma membrane microdomains enriched in gangliosides, play a significant role in regulating signaling pathways and cancer progression [57–59]. Previous studies have reported that the gangliosides GD3 and GM1 are associated with lipid raft formation [36, 37]. In this study, we found that ganglioside GD2 could also influence lipid raft formation. HGF/c-Met signaling, which is localized in lipid rafts, correlates with the tumor microenvironment [60]. In general, HGF is required to activate the HGF/MET pathway. Interestingly, we showed that B3GALT4 knockdown induces c-Met signaling pathway activation in an HGF-independent manner. The PI3K/AKT/mTOR pathway is common in cancers and plays a key role in regulating the tumor microenvironment [61]. The serine/threonine kinase AKT is a representative signaling protein associated with lipid rafts [62–64]. The IFN-γ/Stat1/IRF-1 axis plays an essential role in the communication between the tumor and the microenvironment [65]. IRF-1, a crucial transcription factor in the IFN-γ signaling pathway, inhibits cell proliferation and promotes immune cell infiltration [66, 67]. The current study showed that B3GALT4 overexpression promoted CXCL9 and CXCL10 expression via AKT/mTOR/IRF-1 signaling. Thus, the c-Met/AKT/mTOR axis may represent a promising therapeutic target in NB.

Rescue experiments demonstrated that blocking lipid rafts with MβCD reversed the inhibitory effect of B3GALT4 knockdown on tumor progression and the tumor microenvironment, indicating that B3GALT4 exerted its function through lipid rafts in NB. Monoclonal antibodies targeting GD2 have been applied clinically to high-risk NB with significant success. Nonetheless, a large number of patients have no obvious response to anti-GD2 mAbs, which is attributed to the low number of immune cells in the tumor microenvironment. Studies have demonstrated that lipid raft inhibitors inhibit tumor cell migration, invasion, and angiogenesis [68, 69]. However, combination therapy with lipid raft inhibitors and immune checkpoint blockers has not been reported. Our results showed that the combination of anti-GD2 immunotherapy with MβCD significantly enhanced chemokine CXCL9/10 expression and CD8^+^ T-cell infiltration, suggesting that combination therapy significantly enhances the response to anti-GD2 immunotherapy in NB.

Our study has several limitations. Considering the heterogeneity of the tumor microenvironment, whether B3GALT4 also modulates the recruitment of other immune cells remains unknown. Given that gangliosides are also involved in T-cell activation, the study of the direct effect of B3GALT4 on the ganglioside composition in T cells is limited.

## Conclusion

**Fig. 9:**
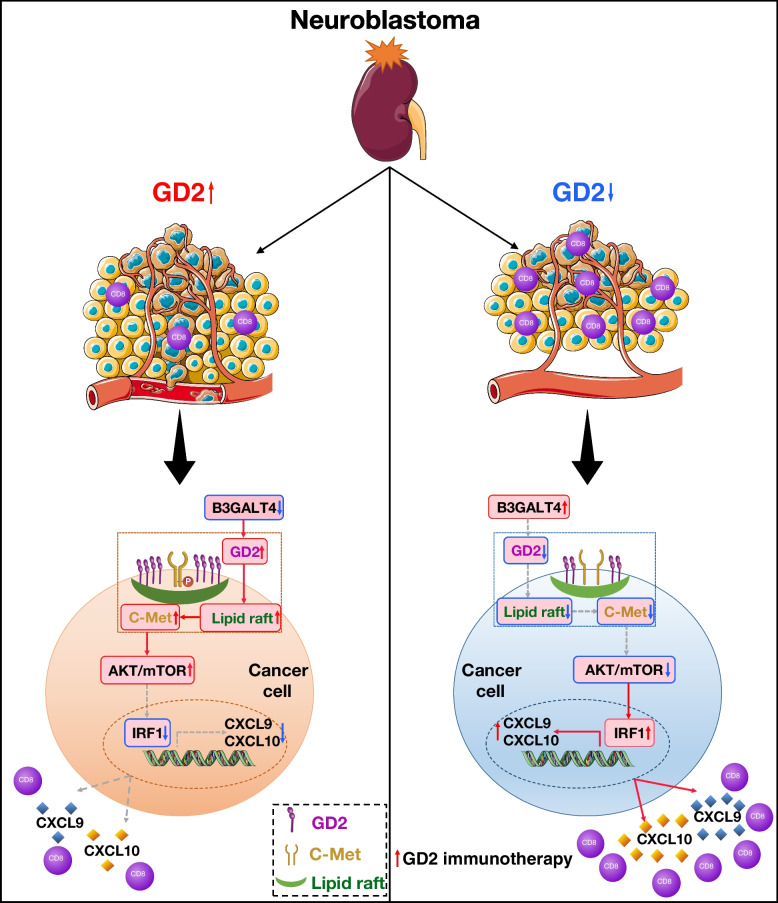
Schematic representation of this study. B3GALT4 overexpression promotes CXCL9 and CXCL10 expression and secretion via the inhibition of c-Met signaling in the lipid raft, accompanied by the downstream Akt/mTOR/IRF-1 signaling pathway. The secreted chemokines recruit CD8+ T lymphocytes to the tumor microenvironment. In particular, lipid raft inhibitor combined with anti-GD2 mAb significantly suppressed tumor growth in the 9464D-derived tumor xenografts in vivo

In conclusion, the present study characterized a tumor-intrinsic function of B3GALT4 in remodeling the TME of NB (Fig. 9). B3GALT4 overexpression inhibited lipid raft formation by downregulation the ganglioside GD2, which further increased the production of CXCL9 and CXCL10 to recruit CD8^+^ T cells via the c-Met/AKT/mTOR/IRF-1 pathway. Combining a lipid raft inhibitor with an anti-GD2 mAb significantly improved T-cell-mediated antitumor effects. Together, our study uncovered the critical mechanism of B3GALT4-mediated tumor immunity, which is attraction of CD8^+^ T cells, and our results indicate that blockage of lipid rafts is a potential therapeutic target for NB.

## Supplementary Information

**Additional file 1: Supplementary Table S1.** Demographic data and patient findings.

**Additional file 2: Supplementary Table S2.** The primary antibodies utilized for western blot analysis, immunochemistry, flow cytometry, and immunofluorescence.

**Additional file 3: Supplementary Table S3.** The shRNA targeting sequences for mouse B3GALT4 gene.

**Additional file 4: Supplementary Table S4.** Primer sequences utilized for real-time PCR in the present study.

**Additional file 5: Fig. S1.** MβCD plus anti-GD2 treatment inhibits tumor growth in vivo. A, Schematic of MβCD and anti-GD2 treatments. B, Representative images of xenograft 9464D tumors with different treatments. C, The tumors were removed for weight analysis 21 days after the beginning of treatments. D, Tumor growth curves of subcutaneous 9464D cells with different treatments. **Fig. S2.** Monitoring of mouse weight during the experiment.

## Data Availability

The publicly available data are provided in TARGET and GEO databases.

## Abbreviations

TME: Tumor microenvironment
NB: Neuroblastoma
B3GALT4: Beta-1,3-galactosyltransferase-4
EMT: Epithelial-mesenchymal transition
GEM: Glycolipid-enriched microdomain
GN: Ganglioneuroma
IHC: Immunohistochemistry
DAB: Diaminobenzidine
DMEM: Dulbecco’s modified eagle’s medium
FBS: Fetal bovine serum
P/S: Penicillin-streptomycin
qRT-PCR: Quantitative real-time PCR
MβCD: Methyl-β-cyclodextrin
HGF: Hepatocyte growth factor
ELISA: Enzyme-linked immunosorbent assay
GSEA: Gene set enrichment analysis
OS: Overall survival
PFS: Progression-free survival
EFS: Event-free survival
RFS: Relapse-free survival
IFN-γ: Interferon-γ
IRF-1: Interferon regulatory factor-1
GT: Glycosyltransferase
