# Supplementary material for: B3GALT4 remodels the tumor microenvironment through GD2-mediated lipid raft formation and the c-met/AKT/mTOR/IRF-1 axis in neuroblastoma
Source: J Exp Clin Cancer Res. 2022 Oct 25;41:314. doi: 10.1186/s13046-022-02523-x (PMC9594894; doi:10.1186/s13046-022-02523-x)
Supplement: Supplementary file 1 — Additional file 1: Supplementary Table S1. Demographic data and patient findings. [file 13046_2022_2523_MOESM1_ESM.docx]

**Table S1. Demographic data and patient findings**

| Characteristic | Patient number (%) |
| --- | --- |
| Total | 81 |
| Sex |  |
| Male | 33(40.7%) |
| Female | 48(59.3%) |
| Age (months) |  |
| <18 | 28(34.6%) |
| ≥18 | 53(65.4%) |
| INSS stage |  |
| 1 | 17(21.0%) |
| 2 | 5(6.2%) |
| 3 | 18(22.2%) |
| 4 | 39(48.1%) |
| 4s | 2(2.5%) |
| Risk group |  |
| LR | 22(27.2%) |
| IR | 21(25.9%) |
| HR | 38(46.9%) |
| MYCN status |  |
| Amplification | 12(14.8%) |
| Not amplification | 69(85.2%) |
| Shimada |  |
| Favorable histology | 53(65.4%) |
| Unfavorable histology | 28(34.6%) |
| Bone marrow metastasis |  |
| Yes | 35(43.2%) |
| No | 46(56.8%) |
| LDH (U/L) |  |
| ≤295 | 16(19.8%) |
| 295–500 | 27(33.3%) |
| 500–1500 | 27(33.3%) |
| >1500 | 9(11.1%) |
| None | 2(2.5%) |
| NSE (ng/l) |  |
| ≤25 | 16(19.7%) |
| 25–100 | 23(28.4%) |
| >100 | 42(51.9%) |
| VMA (mg/24 h urine) |  |
| ≤13.6 | 4(4.9%) |
| >13.6 | 48(59.3%) |
| None | 29(35.8%) |
| Follow-up |  |
| Alive | 64(79.1%) |
| Dead | 17(20.9%) |
| Relapse |  |
| Yes | 20(24.7%) |
| No | 57(70.4%) |
| None | 4(4.9%) |
