# Supplementary material for: B3GALT4 remodels the tumor microenvironment through GD2-mediated lipid raft formation and the c-met/AKT/mTOR/IRF-1 axis in neuroblastoma
Source: J Exp Clin Cancer Res. 2022 Oct 25;41:314. doi: 10.1186/s13046-022-02523-x (PMC9594894; doi:10.1186/s13046-022-02523-x)
Supplement: Supplementary file 2 — Additional file 2: Supplementary Table S2. The primary antibodies utilized for western blot analysis, immunochemistry, flow cytometry, and immunofluorescence. [file 13046_2022_2523_MOESM2_ESM.doc]

**Table S2. The primary antibodies utilized for western blot analysis, immunochemistry, flow cytometry, and immunofluorescence.**

| Antibody | Company | Catalog Number | Dilution |
| --- | --- | --- | --- |
| GD2 | BD Biosciences | 554272 | 1:1000 |
| GD2 | BD Biosciences | 554272 | 1:100 |
| GD2 | BD Biosciences | 554272 | 5μg |
| B3GALT4 | Abcam | ab169759 | 1:1000 |
| B3GALT4 | Abcam | ab169759 | 1:200 |
| c-Met | Abcam | ab51067 | 1:1000 |
| p-c-Met | Cell Signaling Technology | 3077T | 1:1000 |
| Caveolin-1 | Cell Signaling Technology | 3267S | 1:1000 |
| Caveolin-1 | Cell Signaling Technology | 3267S | 1:400 |
| Caveolin-1 | Cell Signaling Technology | 3267S | 1:200 |
| AKT | Santa Cruz Biotechnology | sc-5298 | 1:1000 |
| p-AKT | Cell Signaling Technology | 4060S | 1:1000 |
| mTOR | Cell Signaling Technology | 2983T | 1:1000 |
| p-mTOR | Cell Signaling Technology | 2971S | 1:1000 |
| IRF-1 | Cell Signaling Technology | 8478T | 1:1000 |
| β-actin | Cell Signaling Technology | 4970 | 1:1000 |
| CD8 | Abcam | ab209775 | 1:2000 |
| Ki-67 | Abcam | ab16667 | 1:200 |
| MIG/CXCL9 | Abcam | ab137792 | 1:1000 |
| MIG/CXCL9 | Abcam | ab137792 | 1:300 |
| IP10/CXCL10 | Abcam | ab8098 | 1:1000 |
| IP10/CXCL10 | Abcam | ab8098 | 1:400 |
| FITC-CD3 | BD Biosciences | 553062 | 1:100 |
| APC-Cy7-CD4 | BD Biosciences | 552051 | 1:200 |
| PE-Cy7-CD8 | BD Biosciences | 552877 | 1:200 |
| APC-Rat-IgG | BD Biosciences | 560720 | 1:10 |
