# Supplementary material for: B3GALT4 remodels the tumor microenvironment through GD2-mediated lipid raft formation and the c-met/AKT/mTOR/IRF-1 axis in neuroblastoma
Source: J Exp Clin Cancer Res. 2022 Oct 25;41:314. doi: 10.1186/s13046-022-02523-x (PMC9594894; doi:10.1186/s13046-022-02523-x)
Supplement: Supplementary file 3 — Additional file 3: Supplementary Table S3. The shRNA targeting sequences for mouse B3GALT4 gene. [file 13046_2022_2523_MOESM3_ESM.doc]

**Table S3. The shRNA targeting sequences for mouse B3GALT4 gene.**

| Gene | Sequences (5’-3’) |
| --- | --- |
| MouseB3GALT4-shRNA1 | CCTACTACTGGTGATCATCTG |
| MouseB3GALT4-shRNA2 | AGACAACAGCTATCCATGAAG |
| MouseB3GALT4-shRNA3 | GGGCACAGGATATGTACTGTC |
