# Supplementary material for: B3GALT4 remodels the tumor microenvironment through GD2-mediated lipid raft formation and the c-met/AKT/mTOR/IRF-1 axis in neuroblastoma
Source: J Exp Clin Cancer Res. 2022 Oct 25;41:314. doi: 10.1186/s13046-022-02523-x (PMC9594894; doi:10.1186/s13046-022-02523-x)
Supplement: Supplementary file 4 — Additional file 4: Supplementary Table S4. Primer sequences utilized for real-time PCR in the present study. [file 13046_2022_2523_MOESM4_ESM.doc]

**Table S4. Primer sequences utilized for real time PCR in the present study.**

| Gene | Forward primer (5’ to 3’) | Reverse primer (5’ to 3’) |
| --- | --- | --- |
| Mouse-B3GALT4 | CTCCTGGCGGTCCTACTACT | CCACCACAGGCATGAGAGTT |
| Mouse-GAPDH | GGTATGACAACGAATTTGGC | GAGCACAGGGTACTTTATTG |
