# Supplementary material for: B3GALT4 remodels the tumor microenvironment through GD2-mediated lipid raft formation and the c-met/AKT/mTOR/IRF-1 axis in neuroblastoma
Source: J Exp Clin Cancer Res. 2022 Oct 25;41:314. doi: 10.1186/s13046-022-02523-x (PMC9594894; doi:10.1186/s13046-022-02523-x)
Supplement: Supplementary file 5 — Additional file 5: Fig. S1. MβCD plus anti-GD2 treatment inhibits tumor growth in vivo. A, Schematic of MβCD and anti-GD2 treatments. B, Representative images of xenograft 9464D tumors with different treatments. C, The tumors were removed for weight analysis 21 days after the beginning of treatments. D, Tumor growth curves of subcutaneous 9464D cells with different treatments. Fig. S2. Monitoring of mouse weight during the experiment. [file 13046_2022_2523_MOESM5_ESM.pdf]

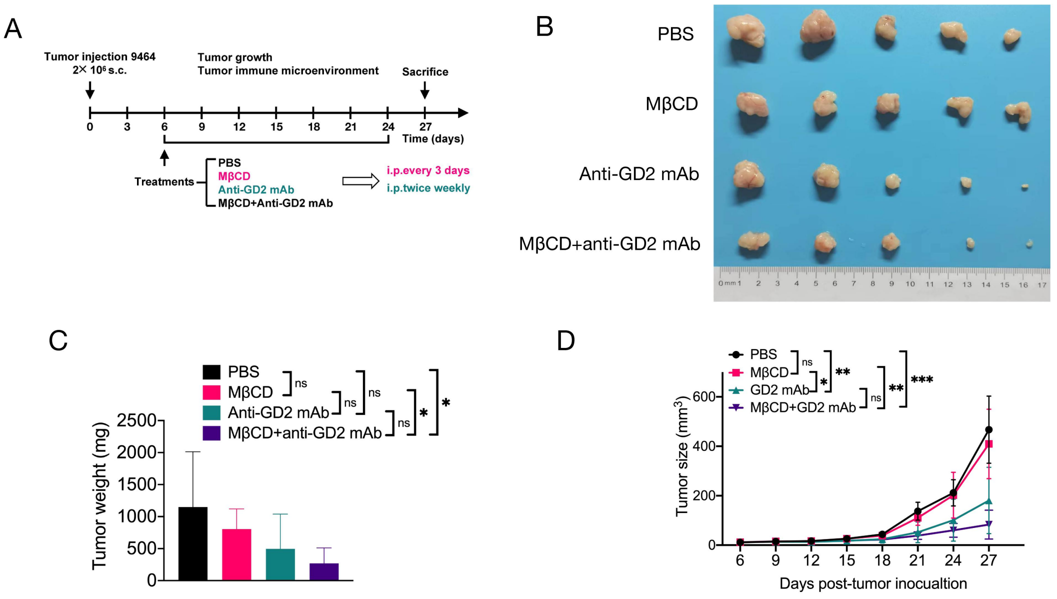

Fig.S1 **MβCD plus anti-GD2 treatment inhibits tumor growth *in vivo*.** (**A**) Schematic of MβCD and anti-GD2 treatments. (**B**) Representative images of xenograft 9464D tumors with different treatments. (**C**) The tumors were removed for weight analysis 21 days after the beginning of treatments. (**D**) Tumor growth curves of subcutaneous 9464D cells with different treatments.

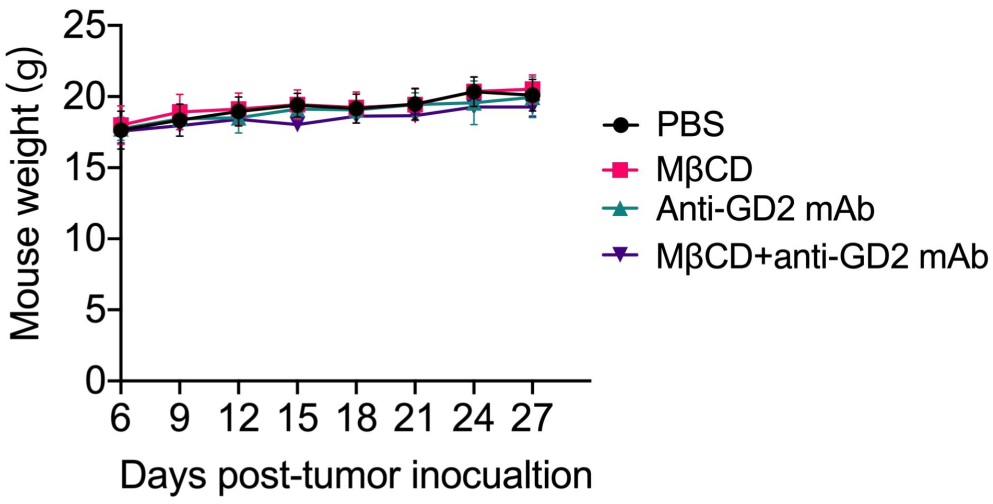

Fig.S2 **Monitoring of mouse weight during the experiment**.
